# Investigation of Hybrid Methods for Elimination of Brilliant Blue Dye from Water Phase Using Various Nanomaterials Combined with Activated Sludge and Duckweed

**DOI:** 10.3390/nano11071747

**Published:** 2021-07-02

**Authors:** Paweł K. Zarzycki, Lucyna Lewandowska, Bożena Fenert, Krzysztof Piaskowski, Janusz Kobaka

**Affiliations:** 1 Faculty of Civil Engineering, Environmental and Geodetic Sciences, Koszalin University of Technology, 75-453 Koszalin, Poland; lucyna.lewandowska@tu.koszalin.pl (L.L.); bozena.fenert@gmail.com (B.F.); Krzysztof.piaskowski@tu.koszalin.pl (K.P.); 2 Faculty of Geoengineering, University of Warmia and Mazury in Olsztyn, 10-720 Olsztyn, Poland; janusz.kobaka@uwm.edu.pl

**Keywords:** micropollutants, wastewater, microanalysis, chemometrics, duckweed, cyclodextrin, activated sludge, hybrid nanomaterials

## Abstract

The main goal of this experimental work is screening of different natural and synthetic nanomaterials and biopolymers that may improve elimination of stable micropollutants from water phase. In this work, as a target chemical acting as the micropollutant molecule, the Brilliant Blue (BB) dye was selected. We tested different active matrices dispersed in water phase including activated carbon (AC), lyophilized graphene oxide (GO), β-cyclodextrin (CD), raw dandelion pappus (DP), microcrystalline cellulose(MC), and raw pine pollen (PP), as well as two types of Egyptian Blue mineral pigments (EB1 and EB2). Graphene oxide and Egyptian Blue nanomaterials were synthesized in our laboratory. We investigated potential application of such nanoparticles and biopolymer conglomerates as additives that may tune the activated sludge (AS) microorganisms or duckweed water plant (DW) and increase efficiency of micropollutants removal from wastewater. Studied nanomaterials/biopolymers were used in two different experimental modes involving real activated sludge microorganisms (24 h experiment) as well as duckweed plant (16 day experiment). Quantitative data of BB were obtained using microfluidic type device based on micro-TLC plate. This approach enabled direct determination of target component without sample pre-treatment like pre-concentration or pre-purification. Within single analytical run calibration line, retention standard spots (methyl red) and multiple samples were analyzed simultaneously. Due to the multivariate nature of these experiments, quantitative data were explored with chemometric tools including AHC (agglomerative hierarchical clustering), PCA (principal component analysis), and FA (factor analysis). Experimental data and multivariate calculations revealed that BB is strongly resistant on biodegradation, however, inclusion complexes formation with β-cyclodextrinmay induce degradation of this dye in the presence of duckweed. It is hoped that results of our experimental work can be used for designing of future experiments for fast screening of different additives and improvement of technological processes, focusing on purification of sewage and water from micropollutants.

## 1. Introduction

Over the last decade there has been a growing interest in the development of non-expensive hybrid technologies, combining nanoparticles generated from recycled waste materials that may work as selective media enabling micropollutants removal from wastewater. This is a complex task, and therefore, a number of different approaches to this problem were proposed and studied. The main issue is that micropollutants form a highly non-homogenous mixture and are present at different concentrations level, usually ranging from pg to ng per liter. They can be chemically resistant, but even under particular conditions (e.g., UV radiation and/or high temperature) some bioactive decomposition products may be generated. In addition, the problem is that micropollutants have a wide range of polarity that may significantly reduce an adsorption ability of given materials [1,2,3,4].

Organic colorants are commonly introduced to water environment by humans, on a global scale. Such chemicals are key reagents and/or final products for various industries producing food, textiles, leather, cosmetics, plastics, electronics, and printer inks. Moreover, in large amount they are present in civil engineering constructions like buildings or marine vessels and can be easily emitted to environment during, e.g., recycling processes. It has been estimated that only the textile industry may consume around 1–3 × 10^5^ tons of synthetic colorants each year. It should be highlighted that in spite of non-complicated detection, the elimination of these xenobiotics from wastewater and surface water ecosystems may be a difficult task due to their high polarity and resistant aromatic chemical structure. The most important issue is that these chemicals may exhibit long term toxicity and work as endocrine disrupting compounds or have mutagenic/carcinogenic activity for given organisms [5,6].

Azo-dyes and triarylmethane dyes are presently considered as the most important classes of colorants due to their versatility and wide variety of color shades available. Some of them can be present in solutions as neutral, mono, or bivalent negatively charged ions [7]. Industrial scale production of these chemicals is easy and non-expensive, therefore, such colorants have been used excessively in industries worldwide [8,9]. Particularly, Brilliant Blue FCF (BB) has been extensively used as a component of food products like sweets, dairy products, and drinks. Recently, due to a potential carcinogenic effect that was documented during a tar-induced tumor study involving rats, this artificial colorant was banned in several European countries [10].

The consequence of the chemical structure of Brilliant Blue FCF is high polarity and solubility in water environment [5]. This colorant is fairly stable under environmental conditions and therefore, a number of selective or non-selective approaches were tested for removal of this micropollutant from water phase. Some of the methods are complex and can be considered as hybrid processes. In the past, BB was investigated as a target component of biodegradation processes performed by the cyanobacteria (*Anabaena flos-aquae* UTCC64, *Phormidiumautumnale* UTEX1580, and *Synechococcus* sp. PCC7942) or enzymatic decolorization processes by acidic horseradish peroxidase [11,12]. However, using these approaches this compound has been found to be difficult for removal with high rate from studied matrices [11,12]. The most efficient seems to be, so far, sorption processes involving e.g., carbons in different forms or iron-modified natural clay products [13,14]. Literature data indicate that Brilliant Blue FCF does not accumulate in plants or animals but degrades slowly in the environment [15]. As an alternative, more complex oxidative degradation has also been studied, however, this process may result with synthesis of number of degradation products, which are not easy to detect because they are colorless [16].

The main concept of this study is fast screening of the elimination process of Brilliant Blue colorant from water phase using complex systems involving activated sludge microorganisms and duckweed water plant. This work was performed in the presence of complex matrices dispersed in water phase including activated carbon, lyophilized graphene oxide, β-cyclodextrin, raw dandelion pappus, microcrystalline cellulose, and raw pine pollen, as well as two types of Egyptian Blue mineral pigments. In such a multivariate experiment various elimination processes of BB are possible, in principle. The main goal of the proposed experimental work is to investigate a potential application of such nanoparticles and biopolymer conglomerates as additives that may tune the activated sludge microorganisms or duckweed water plant and increase efficiency of micropollutants removal from wastewater. It should be noted that some of the selected additives are frequently present in the environment in large amounts, like pine tree pollen, duckweed plant, or dandelion pappus biomass. The second aim of the present work is to demonstrate applicability of simple microfluidic type device based on micro-TLC plate for direct quantitative determination of target component without sample pre-treatment, particularly pre-concentration and pre-purification.

## 2. Materials and Methods

### 2.1. Chemicals

Graphene oxide (GO) was previously synthesized in our laboratory using a modified Hummer’s method according to the detailed protocol reported in [17]. Egyptian Blue mineral pigment was recently prepared by our team accordingly to the methodology reported in literature [18]. β Cyclodextrin was obtained from Merck (Darmstadt, Germany); Cellulose microcrystalline (≅50 µm particle size) from Sigma-Aldrich (Saint Louis, MO, USA); Activated Carbon (Norit SA Super) was obtained from Sigma-Aldrich (Saint Louis, MO, USA); Brilliant Blue E133 produced by Roha Europe (Valencia, Spain). Moreover, methyl red—ACS reagent crystalline—was purchased from (Sigma Aldrich, Saint Louis, MO, USA), 100% methanol (LiChrosolv for Liquid Chromatography) and HPTLC 60RP18 WF_254_S glass coated plates were from Merck (Darmstadt, Germany).

### 2.2. Quantitative Micro-TLC Chromatography

**Figure 1:**
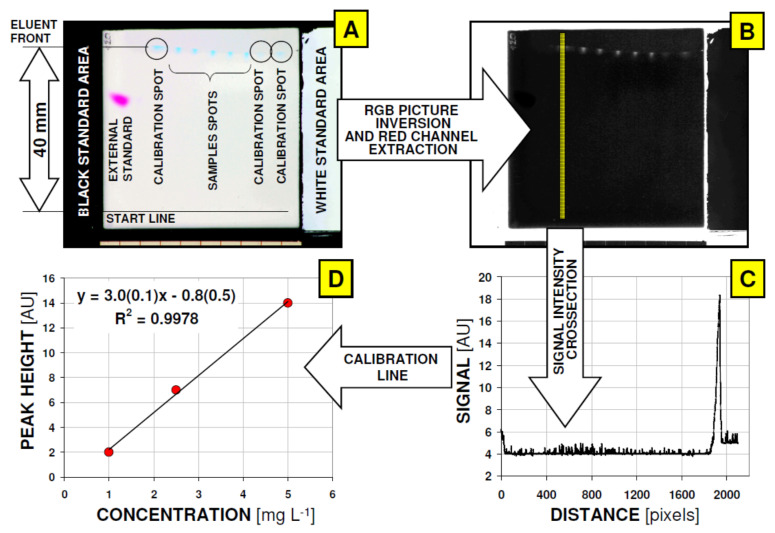
Applied sequence of quantification protocol for direct determination of Brilliant Blue dye in reaction mixtures using microfluidic analytical device based on micro-TLC plate. General view and spots arrangements of micro-TLC analytical device (**A**); to increase the contrast of spots for visual evaluation and printing a global balance filters were applied); converted picture prepared for spots quantification (**B**); to increase the contrast of spots for visual evaluation and printing a global balance filters were applied), Lane cross-section for given calibration spot (**C**); signal intensity data were derived from raw red channel without contrast enhancement); Calibration plot for individual m-TLC plate (**D**). External standard spot (Methyl Red; chromatographed mass 100 ng/spot) was used for accuracy of retention process and quantitative data validation.

Microfluidic device based on glass coated planar chromatographic plate was developed in temperature controlled micro-TLC chamber as described previously [19].For analytes determination, direct application of reaction mixture was performed (2 μL samples) without further pre-purification or pre-concentration. Developing distance was set at 40 mm due to migration of target component close to the mobile phase front. This enabled quantification of BB using peak base line reference. Additionally, external standards methyl red (100 ng/spot) was chromatographed simultaneously in a separate lane within given micro-TLC plate (Figure 1). All chromatographic runs involved 100% methanol as the mobile phase and HPTLC 60RP18 WF_254_S glass coated plates as the stationary phase. All runs were performed at 20.0 °C.

### 2.3. Biological Materials

Pollen was collected from pine trees in the Koszalin (Poland) area in May 2017. This material was air dried, sieved, and stored in a sealed glass container until the experiment was performed.

Dandelion Pappus was collected at Szczecinek (Poland, May 2020) area, air dried, and stored in glass container. For experiment purposes, the pappus feathers were separated from dandelion seeds.

**Figure 2:**
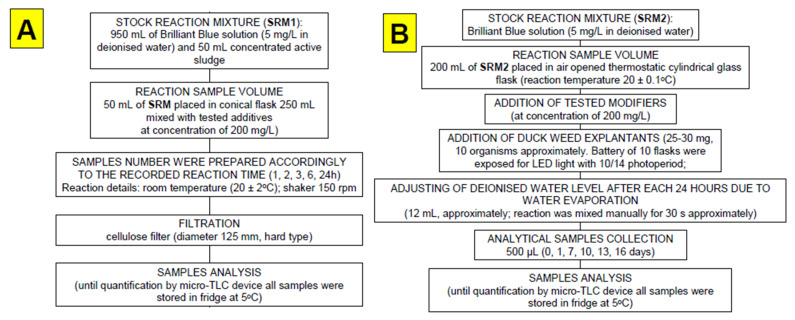
Sequence and protocol details of 24 h (**A**) and 16 day (**B**) tests.

Activated sludge samples were collected from “Jamno” Wastewater Treatment Plant (“J”WTP; located within Koszalin city area, Poland), and we used biomass from the activated sludge pumping station. Particularly, concentrated excessive sludge was collected from the bottom of the secondary settling tank. This material was additionally centrifuged (using MPW 350, Warszawa, Poland) for 10 min. at 700 rpm and an appropriate volume of this stock biomass was mixed with the remaining sample components for 24 h biodegradation experiment (according to the scheme presented in Figure 2A).

Duckweed biomass used in this work was part of the samples that were collected on 11 October 2012 by PZ from the surface water ecosystem that is part of Dzierżęcinka River passing through Koszalin (N 54° 11.579′ E 016° 11.021′). Until experiment time performed in 2021, duckweed organisms were breeding in a small aquarium (volume 28 L; temperature 20–26 °C; photoperiod: 14h light interval in a 24 h period using 25 W incandescent light bulb) and regularly refilled with tap water. The water container consisted of a natural wood block and was cohabitated with 2–5 fish (*Ancistrusdolichopterus)*, which were fed with common fish food. In September 2015, due to an accidental electric energy shutdown for 11 days, 99% of duckweed plants were lost. However, duckweed population was reconstructed from 2–3 plant organisms that survived. Biodegradation test (16 days) was performed within air opened thermostatic cylindrical glass containers (internal diameter = 40 mm, h = 230 mm) connected to Thermostat Huber (Offenburg, Germany). Detailed protocol was described in scheme presented within Figure 2B.

### 2.4. Synthesis of Egyptian Blue Pigment

This nanomaterial was prepared using sequential heating involving a N 61/H 20 kW oven (Nabertherm GmbH, Lilienthal/Bremen, Germany). Synthesis was performed in air environment under 860 °C and next for 950 °C temperatures. Heating time for given temperature was 4 h and then the pigment material was cooled inside the oven cavity to room temperature for the next 19 h. Overall heating time was 24 h, including 1 h for temperature stabilization starting from room to 860 or 950 °C. Pigment dye samples (labeled as EB1 and EB2) were obtained using different spatial arrangement of raw pigment (860 °C) that was re-heated in 950 °C. Particularly, EB1 material was re-heated at 950 °C directly on fireclay brick base whilst EB2 material was placed within porcelain steamer (ID = 9 cm, approximately).After heating and re-heating stages, all sintered materials were grinded using Retsch Ball Mill S100 (Haan, Germany) set at 450 rpm, 2 × 15 min with rotation changes using two types of grinding balls (10 mm, 30 mm). Details of this process are part of LL PhD thesis and will be published in the future.

### 2.5. Dye Removal Test and Measurements 

Biodegradation test (24 h) was performed using a conical glass (250 mL Erlenmeyer flasks) attached to laboratory shaker Laboshake Gerhardt GmbH 500 (Königswinter, Germany) with shaking speed set at 150 rpm. Zeta potential and particle size were measured using Zeta Potential Analyzer, ZetaPALS Brookhaven Instruments Corporation (Holtsville, NY, USA). This device was working with ZetaPALS particle sizing software- 9kpsdw ver. 2.31(1997) and PALS zeta potential analyzer software ver. 3.16 (1998). pH values were measured using Handylab pH11 (Schott, SI Analytics GmbH, Mainz, Germany) equipped with pH-electrode BlueLine 24 pH (pH 0–14/−5–80 °C/Gel). Measurement of macroparticles (pine pollen, dandelion pappus) size was based on images generated by scanning electron microscopy (SEM; JEOL JSM-5500LV).

### 2.6. Data Acquisition and Analysis

Quantitative data from micro-TLC plates were extracted from unprocessed digital images (acquired by Canon EOS 1100D SLR digital camera and homemade lighting box equipped with UV and visible light sources) using ImageJ software (ver.1.48 Wayne Rasband, National Institutes of Health, Bethesda, MD, USA; http://rsb.info.nih.gov/ij, accessed on 4 February 2017). Digital SLR camera worked with Tamron 55–200 lens and HAMA filter (UV 390/52 mm). Pictures were acquired from a distance of 94.5 cm with the following setup (F16; 1/4s). The acquisition system included a ring of 12 LED lamps (JDR, SMDHLCW-250; 3.5W; 6400 K; 250 Lumens, Sanico Electronics, Warszawa, Poland). Two glass plates coated with carbon soot and magnesium oxide were used as the black and white references. For micro-chromatograms images presented in this work, a global manual balance filter was applied to increase the contrast for spots visual evaluation and printing.

Quantitative data concerning investigated samples were inspected with PCA procedure using XLSTAT XLSTAT-Pro/3DPlot statistical and visualization package (version 2008.2.01) provided by Addinsoft (Paris, France) and working with Microsoft Excel 2002. The appropriateness of multivariate calculations for our data was assessed by performing the Bartlett’s sphericity test.

## 3. Results and Discussion

### 3.1. Problem Overview and Experiment Concept

In our previous works we demonstrated degradation of different endocrine disrupting compounds (EDCs), using protocols involving aerobic batch experiment with activated sludge obtained from the municipal sewage treatment plant [20] as well as duckweed biomass [21]. Moreover, we investigated behavior of activated sludge biomass in the presence of different type of carbon materials including graphene oxide nanoparticles [6]. Our experimental work using Ternes methodology [22,23] demonstrated relatively fast degradation rate of different steroids including estriol and testosterone, whilst equilin was fairly resistant for biodegradation under activated sludge aerobic conditions [20]. Quantitative analysis of these reaction mixtures using temperature-dependent inclusion chromatography (TDIC; column high-performance liquid chromatography with diode array UV-Vis detection) has revealed a number of degradation products generated from the parent estriol and testosterone molecules [24]. TDIC protocols were also applied for analysis of degradation of selected bisphenols in the presence of β-cyclodextrin (β-CD) and duckweed water plant [21]. We studied the potential encapsulation effect and removal efficiency of nontoxic macrocyclic oligosaccharide (β-CD) acting as an encapsulation reagent, which may promote the removal of selected bisphenols (bisphenols A, B, and S) from the liquid phase both with and without the presence of duckweed biomass. Results of multivariate calculations clearly suggested differences in bisphenols behavior in the presence of β-cyclodextrin or β-cyclodextrin/duckweed additives. In our latest work based on activated sludge jar test we confirmed the complex interaction of graphene oxide (GO) nanoparticles with microorganisms that are present in activated sludge working with real wastewater [6]. Univariate measurements including pH, conductivity, TOC, IC, ammonia, and total nitrogen together with multivariate statistics calculations (principal component analysis) clearly revealed that GO may significantly affect the wastewater technological processes investigated.

It should be noted that column chromatographic separation combined with UV-DAD quantification of target analytes can be accurate, but in reality, it is also laborious (column cleaning and conditioning, mobile phase degassing, data acquisition for calibration lines), time consuming (only one sample can be analyzed during one separation run and no parallel samples processing mode can be applied for one HPLC machine), and expensive (typically applied flow rate 1 mL/min generate high solvent usage and cost in case of e.g., acetonitrile component). Most importantly, this type of analysis requires mandatory sample pre-purification from solid particles (to avoid valve and column clogging) and sample solvent replacement due to HPLC mobile phase compatibility (for column chromatographic analysis the same sample solvent/mobile phase composition is required). These issues result in additional evaporation steps and application of solid-phase extraction (SPE) procedure for samples pre-concentration and pre-cleaning, which itself istime consuming and expensive. Therefore, our team is developing an alternative quantitative protocol that is based on microfluidic devices, particularly micro-TLC concept, enabling parallel samples analysis with direct injection of raw materials without samples pre-cleaning and solvent replacement. We have demonstrated that this methodology can be robust, accurate enough for given applications, and sensitive for a number of target analytes and various complex inorganic or biological matrices [25,26,27,28,29,30,31]. Using such analysis, the target components can be efficiently separated from background impurities, including solid particles, and parallel analysis mode is possible. Importantly, this approach enables calibration line generation and samples analysis within one analytical run. In case of a micro-plate with a size of 5 cm × 5 cm, up to 9 individual samples can be processed.

In case of brilliant blue analysis there is an additional problem related to high polarity of this chemical. As consequence, this analyte is strongly adsorbed by chromatographic stationary phase (in case of planar chromatography analyte spot is adsorbed on plate start line) or migrate with solvent front, depends on the mobile phase/stationary phase properties (reversed or normal phase systems). It has been found that BB migration between these states (on start line or close to mobile phase front) results with broad and tailing spots that are inconvenient for quantitative analysis. For our purposes, due to the presence of solid nanoparticles additives in raw samples that are difficult to remove (also by centrifugation) we selected the micro-plate mobile/stationary phase system where BB can migrate with mobile phase front (analyte is non retarded by stationary phase). Such an effect was obtained using 100% methanol as a mobile phase, which additionally was simplified by our quantification system. Total migration distance of analytes were 40 mm. Device and samples arrangement picture as well as all key quantification steps are visualized in Figure 1. According to this protocol, basic validation data for methyl red retention standard (*n* = 20) measured as a peak intensity (PI; peak heights of green channel; 8 Bits resolution) and peak area (PA; number of pixels above baseline) resulted with average PI = 185 ± 37 (relative standard deviation RSD = 5.07%), and PA = 3946 ± 772 (relative standard deviation RSD = 5.11%). Using proposed device setup up to five BB samples can be analyzed together with three points for calibration line and one lane for retention control standard (methyl red).

**Figure 3:**
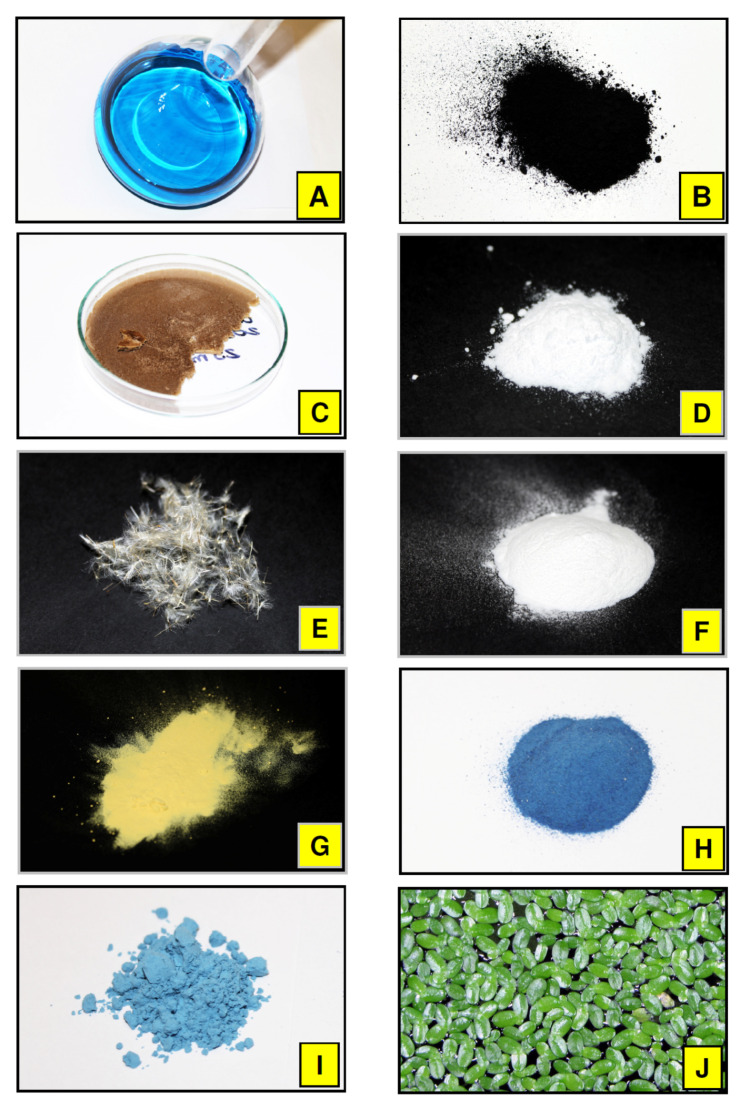
General view of target chemical stock solution (Brilliant Blue 5 mgxmL^−1^; (**A**) and additives studied: active carbon (**B**), lyophilized graphene oxide (**C**), β-cyclodextrin (**D**), raw dandelion pappus without seeds (**E**), microcrystalline cellulose (**F**), raw pine pollen (**G**), Egyptian Blue (synthesis 1 EB1-(**H**); synthesis 2 EB2-(**I**), and duckweed water plant (**J**).

**Figure 4:**
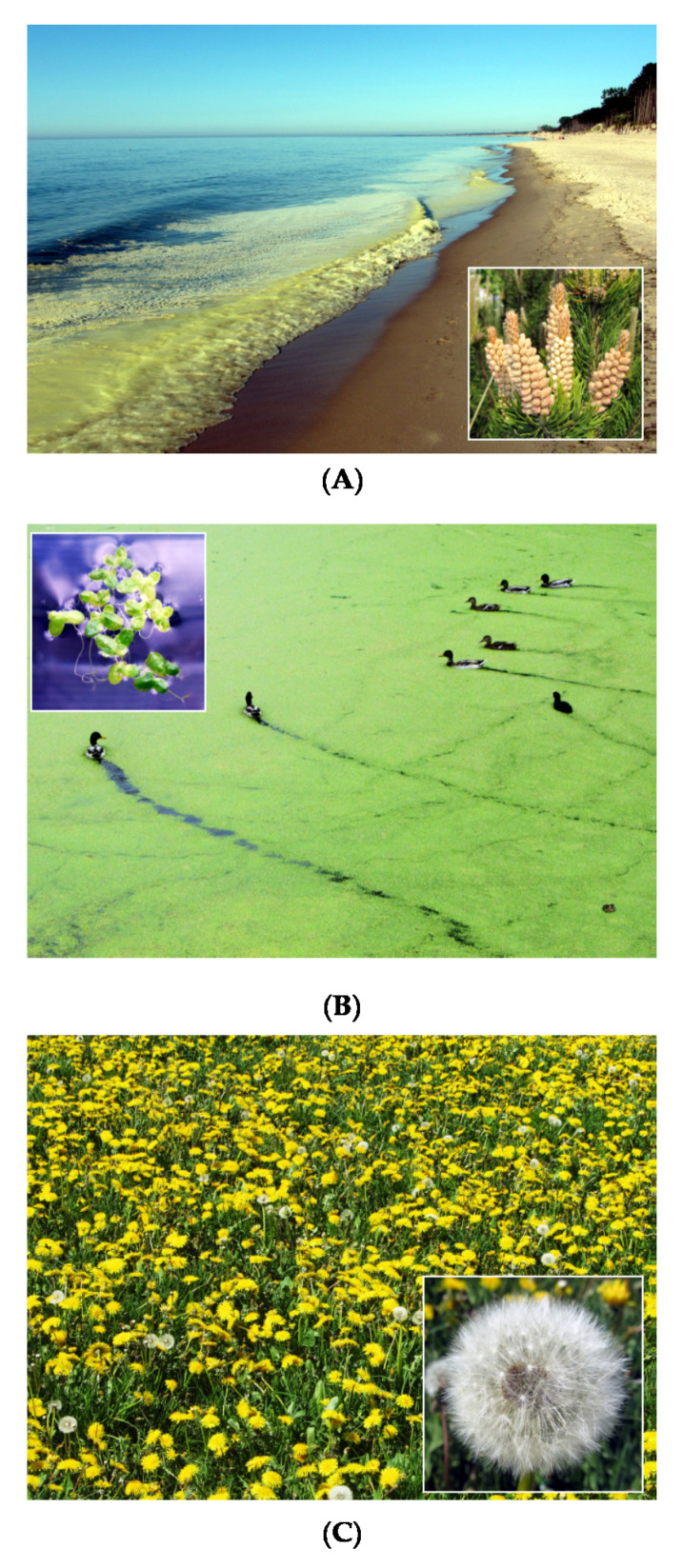
Massive spread of pine pollen biomass in May along Baltic Sea shore (**A**), duckweed (**B**) plants in Poland, dandelion (**C**).

Experimental work proposed in this paper involved a number of various nanomaterials/biopolymers, which were used in two different experimental modes involving real activated sludge microorganisms (24 h experiment) and duckweed plant (16 day experiment). Detailed protocols concerning both experiments are listed in Figure 2A,B. A general view of target component (Brilliant Blue) and additives is present in Figure 3. We have selected these materials due to (*i*) expected different mechanisms that may occur during interaction with BB and active biomass (activated sludge and duckweed), and (*ii*) presence of selected biomaterials in various ecosystems in large amounts (pine tree pollen, duckweed plant, or dandelion pappus biomass; Figure 4). Our analysis revealed that Brilliant Blue compound is negatively charged (zeta potential around −2 mV) in water solutions at pH = 5–6. Generally, adsorption process occurs as a result of electrostatic attraction of ligands or the interaction of intermolecular forces between the non-polar fragments of dye and the adsorbent. This process depends on the nature of the adsorbent surface and can be characterized, e.g., by electrokinetic potential [32].

Possible mechanisms that may increase BB elimination from water phase, mainly involving biodegradation, adsorption, or inclusion complexes formation, can be the following:

(a) activated carbon(AC) Norit SA Super is a common adsorption base that is very efficient but predominantly not selective. Selected in this experiment, AC is characterized by particle size 15 μm (D_50_) and high surface area. This material is produced by steam activation resulting with 1150 m^2^ g^−1^ total surface area (BET). It has been found that the surface charge of activated carbon may be negative in the wide range of pH values ranging from 3.5 to 9.5. This allows efficient electrostatic attraction of target molecules that particularly consists of cationic ligands; however, different molecules can be also efficiently adsorbed on AC due to e.g., dispersion forces. This material is commonly applied in wastewater treatment technologies in different modes and spatial forms or as support for different active molecules [14,33,34,35].

(b) lyophilized graphene oxide (GO) may form individual nanoparticles and its conglomerates in water solution but is chemically and structurally non-homogenic. Physicochemical properties of GO may vary depends on the synthesis type and drying methodology of raw product as well as storage time and conditions (temperature, oxygen contents, UV-Vis light exposition) [17]. We have documented that GO additive may significantly change biological activity of activated sludge microorganisms [6]. Graphene oxide seems to be a promising material to develop new hybrid nanocomposites involving natural polysaccharides for removal of dyes from wastewater [36]. In our experiment, both direct interaction of BB with GO and effects of GO on activated sludge microorganisms can be present.

(c) β-cyclodextrin (CD)belongs to a chemical group called macrocyclic oligosaccharides. In polar solutions like water, this molecule may form stable inclusion complexes (host–guest interactions) and therefore native or derivatized cyclodextrins have a number of practical applications in pharmacy, food chemistry, and analytical chemistry. They may act as the selective removal agents for micropollutants from wastewater [2,21,37,38].

(d) raw dandelion pappus (DP); this organic structure is based on cellulose biopolymer. This structure contains a number of different organic substances changing pappus surface polarity, e.g., waxes. Recently, a dandelion plant was used as a useful matrix in determination of trace elements pollution in various ecosystems [39]. Dandelion pappus biomass is present in large amounts in the environment, can be detected in sediments, and may affect benthic biota [40,41]. Most recently, a number of new applications of such biomaterial were reported, including new supercapacitors or extraction matrix for micropollutants analysis [42,43]. In the proposed experiment, direct adsorption of BB on dandelion pappus biomass and effect on activated sludge microorganisms growing can be expected.

(e) microcrystalline cellulose (MC); this biopolymer based on glucose monomers is complex at any scale. It contains both crystalline and amorphous zones and may adsorb both polar as well as non-polar target chemicals from water phase. It is commonly used in separation science due to fact that it may work both in normal phase and reversed phase mode [44,45].In the proposed experiment MC should act similar to the DP modifier.

(f) raw pine pollen (PP); This multicompartment biological microstructure can be locally present in the natural environment in large amounts. Pollen wall membranes contain highly resistant polymers like sporopollenin. Pollen can also be used as natural microcapsulation particles after isolation of clean sporopollenin exine capsules [46,47,48]. In our experiment, multiple mechanisms can be expected in the presence of pollen in reaction mixture, including physical and chemical adsorption as well as have an effect on activated sludge microorganisms.

(g) Egyptian Blue mineral pigments (EB1 and EB2). Recently, it has been discovered that this ancient mineral pigment may spontaneously delaminate in water and form various nanoparticles. Moreover, it may act as an efficient fluorophore transferring visible light into near infrared wavelengths. This material can be relatively easy produced in different forms in laboratory [18,49,50,51,52].We are expecting interaction of BB nanoparticles containing copper ions with both BB and activated sludge microorganisms.

### 3.2. Detailed Results and Multivariate Data Analysis

Data included in Table 1 clearly indicates that each additive is unique in term of geometry, particle size, and surface physicochemical properties quantified as Zeta potential. All materials used in our research showed a negative value of the zeta potential, which determines the negative surface charge of the molecules, but some of them may have a specific physicochemical and chemical structure, particularly activated carbon, cyclodextrin, and graphene oxide. In our experiments we tested all mentioned above materials to check if they may promote elimination of Brilliant Blue from the water phase. This can be performed by direct interaction with component of interest or indirectly, by affecting the activated sludge microorganisms. Due to the multivariate nature of proposed experiment and multiple elimination mechanisms and/or simultaneous effects expected, quantitative data obtained from 24 h or 16 day experiments were analyzed using chemometrics exploratory tools including PCA, AHC, and FA protocols.

**Figure 5:**
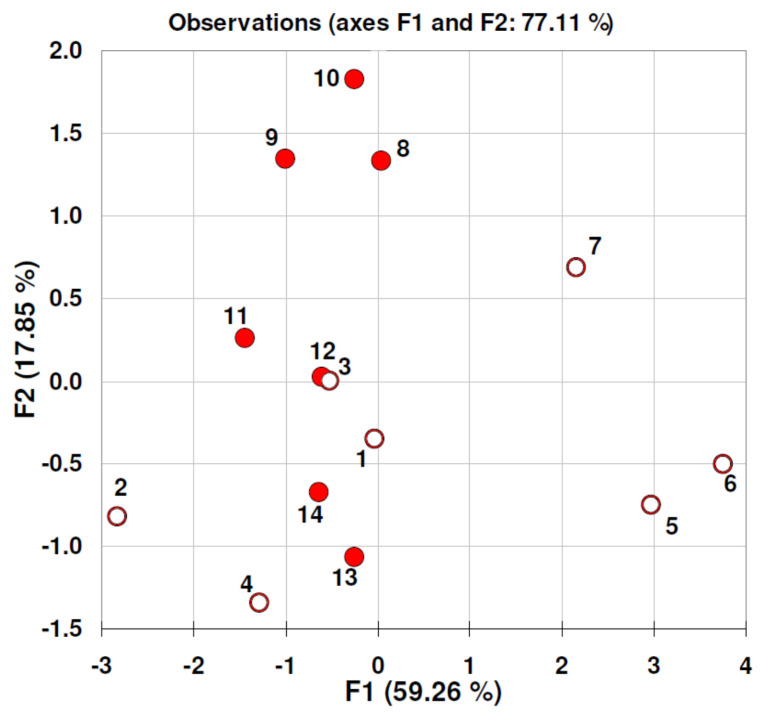
Factor scores plot (principal component analysis) related to 24 h experiment and calculated from data matrix consisting of Brilliant Blue concentrations at different times of experiment duration (variables) and different additives (objects) listed in Table 2. Object labels (circles correspond to samples without activated sludge addition): water BLANK (1), water and graphene oxide GO (2), water and β-cyclodextrin CD (3), water and raw dandelion pappus DP (4), water and microcrystalline cellulose MC (5), water and raw pine pollen PP (6), water and Egyptian Blue mineral pigments EB2 (7), activated sludge AS BLANK (8), activated sludge and graphene oxide GO (9), activated sludge and β-cyclodextrin CD (10), activated sludge and raw dandelion pappus DP (11), activated sludge and microcrystalline cellulose MC (12), activated sludge and raw pine pollen PP (13), activated sludge and Egyptian Blue mineral pigments EB2 (14).

Results of quantitative analysis of BB concentration in studied reaction mixtures for both experiments are present in Table 2 and Table 3. As it was expected, total elimination of BB molecules from studied reaction mixtures was observed in the case of active carbon additive. These data confirmed our previous observation that active carbon may efficiently adsorb BB and different dyes within first 2 h of contact time [14]. In the present experiment BB was completely removed from the reaction mixture within the first hour of contact time with AC. Therefore, this additive was excluded from multivariate calculations. Results of principal component analysis (factor scores plot) are presented in Figure 5. Explored data concerned 16 h experiment setup, which was based on parallel reaction mixtures with additives in the presence and without activated sludge microorganisms. As can be seen, there is no sample (objects) discrimination in term of activated sludge addition type (objects from 1 to 7 without activated sludge; objects from 8 to 14 activated sludge added). Moreover, all objects seem to be randomly distributed in F1/F2 factor space. This strongly suggests that experiment time is too short to register real concentration changes of BB molecules in studied reaction mixtures. Interestingly, under similar experimental conditions (24 h jar test) where wastewater instead of BB molecules was exposed to carbon additives (including graphene oxide), we have observed significant changes in selected physicochemical parameters of both: (*i*) activated sludge (e.g., SSV30 -settled sludge volume after 30 min, SVI—sludge volumetric index and CST—capillary suction time) and (*ii*) processed wastewater (pH, conductivity, and concentration of total nitrogen) [6]. Obtained results have shown that carbon materials, particularly graphene oxide in different forms, may strongly change wastewater processing in the presence of activated sludge microorganisms. Our current results indicate that Brilliant Blue molecules are strongly resistant for short term biodegradation also in the presence of various additives. It may be hypothesized that effective adsorption or chemical reaction between BB and additives studied depends on electrostatic interaction between due ligands and adsorbent surface. According, to data of Zeta potential of additives presented in Table 1, the negative charged surfaces can be expected in water solutions. In case when the adsorbate and adsorbent surfaces have uniform charges, adsorption mechanism may be explained by possible bridging with multivalent cations, which are present in the solution or composition of the adsorbent [32]. On the other hand, the BB molecule can be either neutral or dissociate to a mono or bivalent anions. This process is strongly pH dependent. Our experiments measured pH values of the reaction mixtures that varied from neutral to pH = 3 (graphene oxide additive). Under such conditions, anionic BB dye cannot be adsorbed on additives studied. However, this phenomenon is more complex because Brilliant Blue was adsorbed well on negatively charged active carbon.

**Figure 6:**
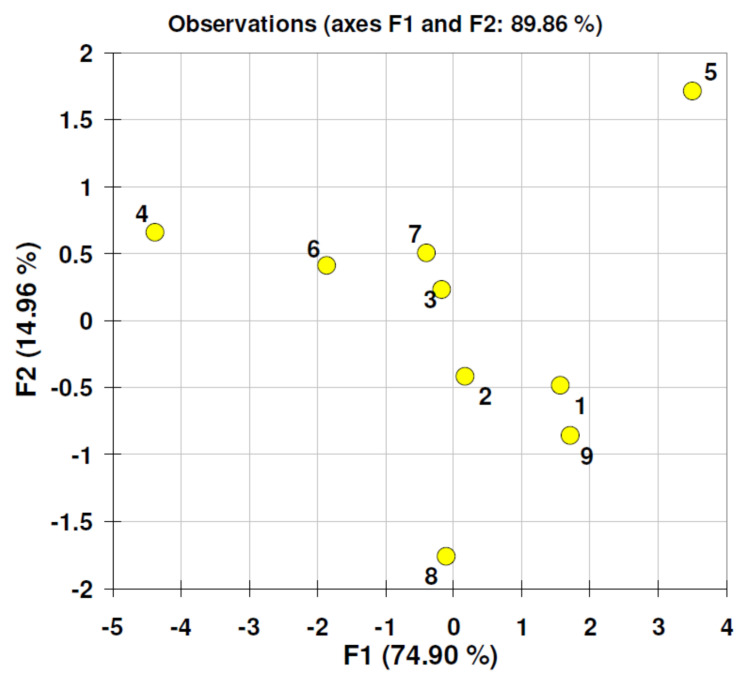
Factor scores plot (principal component analysis) related to 16 day experiment and calculated from data matrix consisting of Brilliant Blue concentrations at different times of experiment duration (variables) and different additives (objects) listed in Table 3. Object labels: water BLANK (1), duckweed water plant DW (2), DW and graphene oxide GO (3), DW and β-cyclodextrin CD (4), DW and raw dandelion pappus DP (5), DW and microcrystalline cellulose MC (6), DW and raw pine pollen PP (7), DW and Egyptian Blue mineral pigments EB1 (8), DW and Egyptian Blue mineral pigments EB2 (9).

**Figure 7:**
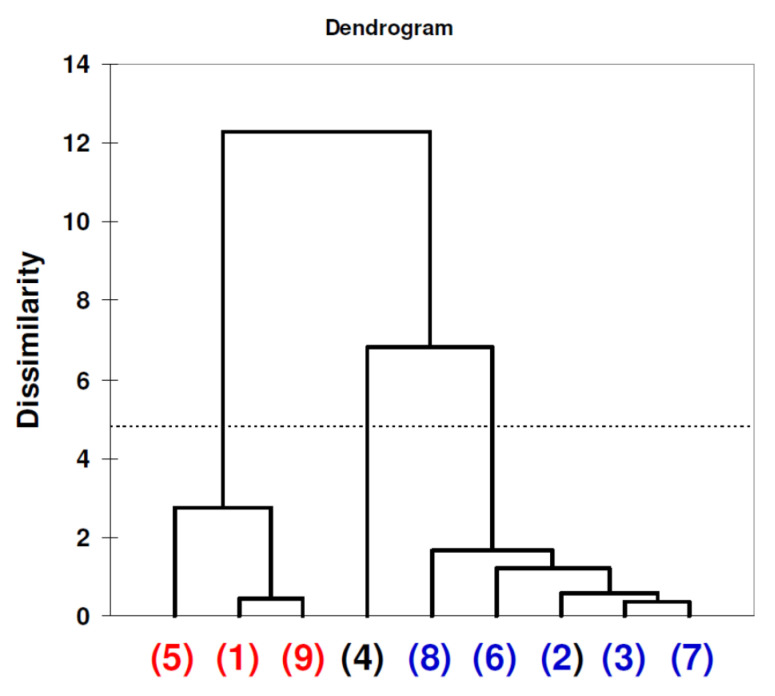
Dendrogram of agglomerative hierarchical cluster analysis involving Ward’s method as the aggregation criterion. The graph represents the clustering of reaction mixtures (BB concentration) with different additives according to experiment time points listed in Table 3 (16 day experiment). Object labels are identical to those listed in Figure 6 caption.

Figure 6 consists of factor scores plot derived from PCA analysis of data matrix generated during the 16 day experiment. These calculations revealed that the 16 day experiment is driven by one dominating factor (F1) and it explains over 74% of the total variability. This was also confirmed by factor analysis (FA) calculations, particularly the Scree plot analysis (eigen values for F1 = 4.241 for F2 = 0.455; cumulative: F1 =70.7 and F1 + F2 = 78.3; graphs not included in this paper). Therefore, analyzing the objects grouping along F1 axis we may see that sample number 4 is separated from remaining objects. This sample was composed of β-cyclodextrin additive. According to quantitative data presented in Table 3, in this reaction mixture systematic decrease of BB concentration was registered. Such clustering is also evident by data analysis performed confirmed another classification tool agglomerative hierarchical clustering (AHC). Data presented in the form of dendrogram (Figure 7) revealed that theβ-cyclodextrin reaction mixture is clustered separately.

## 4. Conclusions

Careful analysis of both PCA and AHC graphs, and considering possible mechanisms of Brilliant Blue interactions with given additives, may strongly indicate that inclusion complexes formation can be an alternative way to remove BB molecules acting as micropollutants from water phase. Moreover, multivariate data analysis resulting with PCA and AHC objects grouping may suggest a potential effect of the given additive on BB elimination, particularly graphene oxide, microcrystalline cellulose, duckweed, pine pollen, and particular form of Egyptian Blue pigment (EB1). However, this must be confirmed by performing different long-term experiments involving wide concentration range of BB, and given additives as well as temperatures and pH. Moreover, to explain potential mechanisms of BB elimination, a number of different target dyes should be processed as references.

## Figures and Tables

**Table 1:** Measured values of particles size and zeta potential for additives studied. Raw materials were suspended in distilled water at concentration of 0.2 mg/mL, followed by 60 min of shaking.

Material	Particle Size Value [nm] (Standard Deviation) *n* = 20	Zeta Potential [mV] (Standard Deviation) *n* = 24
Graphene Oxide	20.253 (7.064)	−20.6 (2.7)
Microcrystalline Cellulose	32.135 (5.816)	−4.9 (2.1)
Pine Pollen	25–35 μm (approximate dimensions based on SEM measurement)	Not available
β-Cyclodextrin	1.53 *	Not available
Dandelion Pappus	Fiber length 3–5 mm Fiber diameter 25 μm (approximate dimensions based on SEM measurement)	Not available
Active Carbon Norit SA	9567 (4632)	−13.3 (2.0)
Egyptian Blue EB1	34.621 (12.683)	−14.9 (6.0)
Egyptian Blue EB2	3.458 (942)	−23.3 (6.5)

* Reference data [45].

**Table 2:** Quantitative data of Brilliant Blue concentration (mg L^−1^) measured in presence of different active matrices during 24 h test (involving activated sludge microorganisms).

Time (Hour)/Matrix Tested *	1	2	3	6	24
BLANK	4.353	5.095	4.918	4.903	5.240
AC	0.000	0.000	0.000	0.000	0.000
GO	3.859	2.076	4.021	3.059	3.303
CD	4.353	5.095	4.470	4.376	4.755
DP	4.353	2.773	3.573	4.903	4.997
MC	6.211	5.899	8.351	5.382	6.952
PP	7.330	5.899	7.027	7.273	6.952
EB2	5.651	6.347	8.351	5.854	4.396
AS BLANK	5.479	5.833	4.921	4.611	3.427
AS + AC	0.000	0.000	0.000	0.000	0.000
AS + GO	4.789	5.833	4.184	3.278	3.427
AS + CD	5.479	5.833	4.184	5.278	2.536
AS + DP	4.789	4.167	5.289	2.278	3.724
AS + MC	3.544	5.197	4.285	5.779	4.241
AS + PP	4.152	3.586	6.100	5.377	4.664
AS + EB2	3.848	4.553	5.737	3.771	5.086

* active carbon (AC), lyophilized graphene oxide (GO), β-cyclodextrin (CD), raw dandelion pappus (DP), microcrystalline cellulose (MC), raw pine pollen (PP), Egyptian Blue mineral pigment (EB2), activated sludge (AS).

**Table 3:** Quantitative data of Brilliant Blue concentration (mg L^−1^) measured in presence of different active matrices during 16 day test (involving duckweed water plant).

Time (Day)/Matrix Tested *	0	1	7	10	13	16
BLANK	4.364	5.346	5.595	4.846	4.709	4.828
DW	4.411	5.202	5.202	4.411	4.016	4.016
DW + AC	4.873	0.000	0.000	0.000	0.000	0.000
DW + GO	4.687	5.392	4.335	3.982	3.982	4.335
DW + CD	3.838	3.648	3.369	3.075	3.046	2.606
DW + DP	5.554	5.113	5.554	5.113	5.995	5.554
DW + MC	4.027	4.353	4.353	3.373	3.700	4.027
DW + PP	4.438	4.774	4.774	3.767	4.103	4.438
DW + EB1	3.700	5.779	4.888	4.294	4.591	3.700
DW + EB2	4.411	5.597	5.202	5.597	4.411	4.806

* active carbon (AC), lyophilized graphene oxide (GO), β-cyclodextrin (CD), raw dandelion pappus (DP), microcrystalline cellulose (MC), raw pine pollen (PP), Egyptian Blue mineral pigment (EB1 and EB2), duckweed water plant (DW).

## Data Availability

The data presented in this study are available on request from the corresponding author.
